# Development and Validation of a Robust and Straightforward LC-MS Method for Measuring Taurine in Whole Blood and Plasma of Dogs and Reference Intervals Calculation

**DOI:** 10.3390/ani15010003

**Published:** 2024-12-24

**Authors:** Tommaso Furlanello, Riccardo Masti, Francesca Maria Bertolini, Valeria Ongaro, Andrea Zoia, Jose Sanchez del Pulgar

**Affiliations:** 1 San Marco Veterinary Clinic and Laboratory, Via dell’Industria 3, 35030 Veggiano, Italy; tf@sanmarcovet.it (T.F.); francesca.bertolini@sanmarcovet.it (F.M.B.); valeria.ongaro@sanmarcovet.it (V.O.); zoia.andrea06@gmail.com (A.Z.); 2 CREA Research Centre for Food and Nutrition, Via Ardeatina 546, 00178 Rome, Italy; jsapuri@hotmail.com

**Keywords:** taurine, method validation, LC-MS/MS, reference interval

## Abstract

Several studies have highlighted the essential role of taurine in maintaining the health of small animals, particularly dogs. Taurine deficiency has been linked to various health issues, especially in certain dog breeds. Therefore, accurately assessing taurine levels in canine blood is crucial for diagnosing and monitoring these conditions. In this study, we present the development of a novel liquid chromatography–tandem mass spectrometry (LC-MS/MS) method for rapidly quantifying taurine concentrations in dog whole blood and plasma. The method was validated according to current guidelines, showing excellent accuracy, precision, and sensitivity across a wide concentration range. Specifically, the limit of quantification was set at 80 nmol/mL for whole blood and 8 nmol/mL for plasma, ensuring the method’s reliability for both matrices. The application of this validated technique to blood samples of healthy dogs allowed for the establishment of reference intervals for taurine concentrations (148 to 495 nmol/mL for whole blood; 42 to 183 nmol/mL for plasma). Due to its robustness and simplicity, this method represents a valuable tool, supporting its routine use in health assessments and enabling more effective monitoring of taurine status in dogs.

## Simple Summary

Taurine is essential for the health of small animals, particularly dogs, as a deficiency can lead to problems like impaired foetal development, retinal degeneration, and cardiomyopathy in certain breeds. This study presents a new validated liquid chromatography–tandem mass spectrometry (LC-MS/MS) method for fast and accurate taurine measurement in canine whole blood and plasma, with limits of 80 nmol/mL and 8 nmol/mL, respectively. The method allowed us to establish reliable reference intervals for healthy dogs and offered a practical and robust tool for routine veterinary use for monitoring taurine concentrations effectively.

## 1. Introduction

Taurine, or 2-aminoethanesulfonic acid, is a non-proteinogenic ß-aminosulfonic acid crucial for cell and tissue functions, including membrane stability and ion channel regulation [1]. Unlike other amino acids, taurine does not participate in protein synthesis but is essential for the heart, brain, retina, and skeletal muscles [1]. It is involved in various biological processes, such as anti-inflammatory responses [2], immune modulation [3], antioxidation [4], bile acid conjugation [5], osmoregulation [6], and calcium homeostasis [7]. Classified as “conditionally essential”, taurine may fall below normal concentrations during growth, pregnancy, adolescence, or disease-related dietary insufficiencies [8]. In companion animals such as dogs and cats, taurine deficiency has been linked to serious health issues, emphasising the need for accurate taurine measurement and adequate dietary intake to maintain health. A primary pathology linked to taurine deficiency is dilated cardiomyopathy, a condition characterised by the dilation and weakening of the heart muscle, particularly the left ventricle, leading to reduced cardiac function [9,10,11]. Although cats require taurine in their diet, dogs can synthesise it from precursor amino acids like cysteine and methionine [12]. Nevertheless, some dog breeds, including Golden Retrievers, American Cocker Spaniels, Newfoundlands, English Setters, Saint Bernards, and Irish Wolfhounds, are predisposed to taurine deficiency and related diseases [9]. Factors contributing to deficiency include low-protein diets, high-fibre diets, beet pulp presence [9], and grain-free diets containing legumes [13]. Monitoring taurine concentrations is especially crucial in dogs on grain-free [9,14] or vegan diets [15], where taurine content may be insufficient. This monitoring also helps manage taurine deficiency in susceptible breeds. The taurine analysis is typically conducted on whole blood and plasma collected in K3EDTA or Lithium Heparin. Whole blood taurine concentration reflects both intracellular taurine (e.g., in leucocytes, platelets, and erythrocytes) and plasma taurine, providing a stable marker of long-term status. Plasma taurine concentration, acting as a reservoir for high-affinity tissues, indicates short-term availability and dietary changes [16]. Whole blood taurine concentration has a lower variability compared to plasma taurine concentration, reflecting more accurately long-term availability. Plasma measurements, however, are useful for reflecting recent taurine changes and short-term needs [17]. Comprehensive taurine status evaluation should include both plasma and whole blood analysis, as whole blood concentrations change gradually and only decrease significantly after prolonged deficiency.

Pacioretty et al. [18] studied taurine depletion and repletion kinetics in cat’s plasma, serum, whole blood, and skeletal muscle. During depletion, plasma, serum, and whole blood showed an initial rapid decrease followed by a slower decline. Upon repletion, plasma levels aligned with skeletal muscle, suggesting plasma only replenishes after tissue requirements are met. Whole blood showed faster repletion but slower depletion compared to plasma. These findings emphasise that measuring both plasma and whole blood taurine concentrations is optimal for clinical assessment.

Taurine measurement in animals is often performed using Liquid Chromatography amino acid analysers [13,14,19,20,21,22]. Despite the advantages of high automation, these methods encounter challenges like interferences, lower specificity, and long analysis durations [23]. Because taurine’s high polarity results in weak retention on standard reverse-phase liquid chromatography columns, pre-column derivatisation is needed [24]. However, derivatisation presents issues, including time consumption, extra costs, and handling complexities. The reaction also requires stabilisation, complicating standardisation. Recent studies have validated LC-MS/MS methods for taurine quantification but still faced challenges ensuring adequate retention time with reverse-phase columns [25,26].

A promising solution involves using Hydrophilic Interaction Liquid Chromatography (HILIC) coupled with tandem mass spectrometry. This method enables rapid, derivatisation-free taurine quantification, with greater efficiency and precision in assessing taurine status in dogs [27]. Therefore, this study aimed to develop and validate a rapid quantification method for taurine in both dog whole blood and plasma without derivatisation by using HILIC and tandem mass spectrometry.

## 2. Materials and Methods

### 2.1. Whole Blood and Plasma Samples

Taurine concentrations in whole blood and plasma were measured from non-haemolysed leftover samples of 120 dogs presented to the San Marco Veterinary Clinic and Laboratory for check-up, diagnostic, or monitoring purposes to establish reference intervals (RIs). All these 120 samples belonged to healthy dogs (i.e., dogs presented for routine annual examination/health screening, elective surgery, blood donor, or pre-breeding examination) or to dogs for which medical attention had been sought for pathologies unrelated to the gastrointestinal tract that could potentially impact taurine absorption. The clinical status of these 120 dogs was confirmed through a combination of physical exams, medical history reviews, and results from blood tests, biochemistry panels, and urinalyses. The dogs were fed mostly with standard commercial diets, with no evidence in their medical records of taurine supplementation. Blood samples were drawn from the jugular, saphenous, or cephalic veins using K3EDTA tubes (Becton-Dickinson; Franklin Lakes, NJ, USA). Whole blood was then centrifuged for 10 min at 3000× *g*, after which plasma was transferred to plastic tubes, frozen at −20 °C, and thawed just before analysis.

### 2.2. Chemicals and Reagents

Acetonitrile and methanol hypergrade for LC-MS were purchased from LiChrosolv^®^ (Merck, Darmstadt, Germany). Taurine analytical standard (purity > 99.9%), Taurine D4 internal standard, pure Bovine Serum Albumin (BSA) and phosphate buffer (PBS) were purchased from Sigma-Aldrich^®^ (Merck, Darmstadt, Germany). Formic acid 98.5% pure was purchased from Supelco (Bellefonte, PA, USA).

### 2.3. Instrumentation and Analytical Conditions

Analysis was performed on a Waters Acquity UPLCTM I-class system, composed of a sample manager, heated column manager and binary solvent manager, coupled to a Xevo TQ-S triple quadrupole Mass-Spectrometry detector (Waters Corp., Milford, MA, USA). Extracted samples were analysed using a Phenomenex Luna^®^ Polar Pesticides Column (100 mm × 2.1 mm, 3 µm) (Phenomenex, Vaerloese, Denmark). Mobile phases consisted of 0.1% formic acid in water (A) and 0.1% formic acid in acetonitrile (B). Analysis was performed using the following elution gradient: 0–1.0 min 97% B; 1.0–3.0 min from 3% to 40% B; 3.0–6.0 min maintained at 40%, then a re-equilibration period at 97% B. Flow rate was set at 0.5 mL/min for the whole analysis and the injection volume was 10 µL. The total run was 10 min. Column and sample temperatures were maintained at 50 °C and 4 °C, respectively. Column, gradient, and mobile phases were specifically optimised to obtain a Retention Time of 2.90, which guaranteed a k′ greater than 2, according to European Pharmacopoeia [28]. The retention factor (k′), which reflects the degree of interaction between the analyte and the stationary phase relative to the mobile phase, was determined using the formula: (t_r_ − t_0_)/t_0_, where t_r_ was the retention time of the analyte and t_0_ the dead time (void time). T_0_ was calculated using the formula (column length × column diameter)/flow rate.

For UPLC-MS/MS analyses, the mass spectrometer was set to the following optimised tune parameters: capillary voltage: 3.2 kV; source temperature: 150 °C; desolvation temperature: 650 °C; source desolvation gas flow: 1000 Lhr^−1^; and source cone gas flow: 50 Lhr^−1^. All data were acquired and processed by Waters MassLynxTM 4.1 software. Detection was obtained by Multiple Reaction Monitoring (MRM) with ESI source in positive ionisation mode. The transition *m*/*z* 126.04 → 44.0 was used for quantification, while transitions 126.04 → 108.0 and 126.04 → 65.0 were used as qualifiers for confirmation purposes. For the Internal Standard, the transition *m*/*z* 130.0 → 48.0 was used for quantification, while transition 130.0 → 112.0 was used as qualifiers.

### 2.4. Optimisation of Analyte Separation: Column Evaluation

Before starting the extraction method development, several columns were tested to achieve the best separation of the analyte. For each test, a 50 ng/mL standard was diluted in the initial mobile phase and tested with columns with different types of functionalisation and HILIC-functionalised columns. Conventional reverse phase columns were Agilent Zorbax SB-C18 1.8 µm (2.1 × 50 mm) (Agilent Technologies, Santa Clara, CA, USA), Agilent SB-AQ 1.8 µm (2.1 × 50 mm) (Agilent Technologies, Santa Clara, CA, USA), Waters ACQUITY UPLC BEH 1.7 µm (2.1 × 50 mm) (Waters Corp., Milford, MA, USA), Waters ACQUITY UPLC^®^ HSS T3 1.8 µm (2.1 × 50 mm) (Waters Corp., Milford, MA, USA), Waters CORTECS UPLC^®^ T3 1.8 µm (2.1 × 50 mm) (Waters Corp., Milford, MA, USA), Waters CORTECS Phenyl 2.7 µm (2.1 × 50 mm) (Waters Corp., Milford, MA, USA), Phenomenex Kinetex Biphenyl 2.6 µm (2.1 × 100 mm) (Phenomenex, Vaerloese, Denmark), Phenomenex Kinetex Phenyl-Hexyl 2.6 µm (2.1×100 mm) (Phenomenex, Vaerloese, Denmark) and Phenomenex Luna^®^ Omega Polar 1.6 µm (2.1 × 100 mm) (Phenomenex, Vaerloese, Denmark) were considered. A gradient ranging from 5% to 60% Methanol in 5 min after a 1-min isocratic run was applied with a flow rate set at 0.5 mL/min. Moreover, the Thermo Scientific Syncronis HILIC 1.7 µm (2.1 × 100 mm) (Thermo Fisher Scientific, Waltham, MA, USA) and Phenomenex Luna^®^ Polar Pesticides 3.0 µm (2.1 × 100 mm) (Phenomenex, Vaerloese, Denmark) were considered as HILIC-functionalised columns. In this case, a gradient ranging from 95% to 40% Acetonitrile in 5 min after a 1-min isocratic run was applied.

### 2.5. Sample Preparation

Whole Blood Samples:

A 0.1 mL aliquot was pipetted into a 10 mL conical centrifuge tube and mixed with 5 mL of pure water. The resulting mixture was vortexed for 5 min and sonicated in an ultrasonic bath for an additional 5 min. Subsequently, 0.1 mL of the solution was transferred to a polypropylene centrifugal tube, diluted in 1 mL of water/methanol (50/50 + 0.1% formic acid) containing 200 ng/mL internal standard, and vortexed for 5 min. A 0.05 mL portion of the new solution was then diluted with 1 mL of water/methanol (50/50 + 0.1% formic acid) and vortex-mixed for a few minutes. The resulting solution was subsequently injected into the LC-MS/MS system.

Plasma Samples:

A 0.05 mL aliquot was pipetted into a 10 mL conical centrifuge tube and mixed with 5 mL of water/methanol (50/50 + 0.1% formic acid) with 10 ng/mL internal standard. The mixture was vortexed for 5 min. Following this, 0.05 mL of the solution was transferred to a polypropylene centrifugal tube, diluted in 1 mL of water/methanol (50/50 + 0.1% formic acid) containing 10 ng/mL internal standard and vortexed for 5 min. The resulting solution was subsequently injected into the LC-MS/MS system.

### 2.6. Method Validation

The reliability and reproducibility of the method were evaluated according to the Guideline on bioanalytical method validation from the European Medicine Agency [29] by assessing linearity, limit of quantification (LOQ), recovery, matrix effect, carryover, selectivity and stability as validation parameters. Moreover, intra- and inter-assay precision and accuracy were evaluated at LOQ, low, mid and high concentrations. As criteria of acceptance, the calibrator standard at LOQ concentration had to be within ±20% from the nominal concentration, while the other had to be within ±15% in each analytical run, and the correlation coefficient r2 had to be ≥0.98.

#### 2.6.1. Standard Solution and Calibrators

The standard preparation for LC-MS/MS analysis was as follows: A stock solution of 1 mg/mL taurine was prepared in 0.2% formic acid in water and kept at 4 °C. Since taurine is an endogenous molecule, obtaining a matrix curve without the analyte is not feasible. Therefore, a decision was made to employ a surrogate matrix-matched calibration curve in the range from 1 µg/mL to 100 µg/mL in PBS-BSA 1%.

#### 2.6.2. Limit of Quantification, Linearity, Selectivity and Carryover

The limit of quantification (LOQ) was established empirically as the lowest concentration whose quantification provided a relative standard deviation lower than 20% and with a signal-to-noise ratio higher than 10. Linearity was studied with an eight-point matrix-matched calibration curve prepared and analysed on three consecutive analytical days. Selectivity was determined by analysing six samples from different subjects spiked at LOQ concentration. Carryover was evaluated in conjunction with the linearity studies by running three consecutive blank samples after the highest calibration point.

#### 2.6.3. Precision, Accuracy, Recovery and Matrix Effect

Precision, accuracy, and recovery were assessed by analysing a pooled set of six randomly selected leftover blood and plasma samples. These samples provided adequate volume for validation and were spiked at four different concentrations: LOQ, low (which corresponds to a threefold LOQ concentration), medium (which corresponds to 50% of the calibration curve) and high (which corresponds to 75% of the calibration curve). For the LOQ concentration in blood, samples were spiked at the concentration of 10 µg/mL, 30 µg/mL for threefold LOQ, 50 µg/mL and 75 µg/mL for 50% and 75% of the calibration curve, respectively. Considering the lower concentration of taurine in plasma compared to whole blood, plasma samples were spiked at 1 µg/mL for the LOQ concentration and 3 µg/mL for 3-fold LOQ, while upper concentrations were maintained at 50 µg/mL and 75 µg/mL for 50% and 75% of the calibration curve, respectively. Each concentration was repeated in five replicates and in three non-consecutive days. Intra- and inter-assay precision was estimated in terms of relative standard deviation, while accuracy was calculated as the percentage of the difference between the expected concentration and the measured one. Recovery was assessed by dividing the taurine responses in whole blood and plasma by those obtained at the same nominal concentration in a water/methanol solution (50/50 with 0.1% formic acid).

The matrix effect was assessed by spiking the samples post-extraction. It was quantified by comparing the responses of taurine in whole blood and plasma to those obtained from solutions prepared at the same nominal concentration in water/methanol (50/50 + 0.1% formic acid). Additionally, for plasma, recovery and matrix effect were conducted at LOQ concentration using worst-case samples, (i.e., lipemic and icteric). Coefficient of variation (CV %) values lower than 20% were considered as the pass acceptance criteria.

#### 2.6.4. Stability

To ensure the stability of the sample, we conducted a comprehensive evaluation by exposing it to various stress conditions. This process included monitoring its stability over time under different temperature settings to simulate potential storage and handling environments. Specifically, we assessed the stability of the analyte in both matrices by using low- and high-Quality-Control samples (three times the LOQ and near the upper limit of quantification), along with an intermediate QC sample. For plasma, the stock concentrations were set at 3, 30, and 50 µg/mL, while for whole blood, concentrations were 30, 50, and 75 µg/mL. Freeze–thaw stability was examined after three cycles of freeze (−20 °C) and thaw (room temperature). Short-term stability was evaluated following storage at room temperature for 4 h. Long-term stability was investigated after storing at −20 °C for 7 days. In all stability tests, the acceptance criteria included a relative standard deviation (SD) and a relative deviation from the target (percentage deviation) within ±15%, based on triplicate measurements.

#### 2.6.5. Reference Interval

The RIs were calculated from 120 samples collected from dogs presented to the San Marco Veterinary Clinic and Laboratory between November 2023 and January 2024. To reflect real-world dietary habits, the study included dogs fed their usual food types and amounts. Taurine content in the diets was not measured, mirroring typical clinical situations where such analysis is rarely performed.

#### 2.6.6. Statistical Analysis

The RIs were calculated using Reference Value Advisor (V2.1), a collection of freeware macroinstructions designed for Microsoft Excel (2016) [30]. Outliers were identified using Dixon–Reed’s and Tukey’s tests, while the distribution of the data was assessed by d’Agostino–Pearson and Anderson–Darling tests (statistical significance *p* ≤ 0.05) [31]. The determination of the RIs for taurine in both matrices were calculated using a parametric statistical method, with a minimum of 120 individuals as recommended by the ASVCP 2011 guidelines [29].

## 3. Results

### 3.1. Column Evaluation

**Figure 1:**
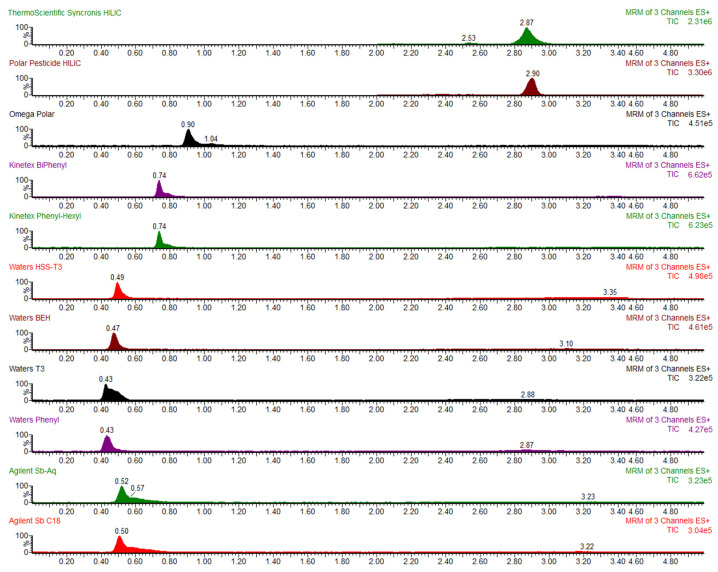
Column evaluation for HILIC columns. An adequate retention from the solvent front is achieved, whereas for all the reverse phase columns the retention time is notably shorter.

Before developing the extraction method, a variety of chromatographic columns was evaluated to identify the most effective column for optimal analyte separation. For each trial, a standard solution of the analyte at 50 ng/mL was injected, and columns with different functionalisation types, including Hydrophilic Interaction Liquid Chromatography (HILIC) phases, were tested to assess their retention and separation efficiencies. As shown in Figure 1, conventional columns exhibited insufficient retention for polar analytes due to their low retention capacity; reverse-phase columns, typically effective for nonpolar to moderately polar compounds, showed limited interaction with the highly polar analyte, resulting in poor retention. This led to k′ values ranging from 0.03 to 1.05 for the 50 mm and 100 mm columns, respectively. Among the 100 mm columns, the Phenomenex Luna^®^ Omega Polar performed best, but still exhibited an inadequate retention time of 0.9 min and a k′ of 1.14. This limited separation capability rendered conventional columns unsuitable for the study’s analytical requirements. In contrast, HILIC-based columns, particularly the Thermo Scientific Syncronis HILIC and the Phenomenex Luna^®^ Polar Pesticides columns, demonstrated superior separation, achieving a retention time of approximately 2.90 min for the analyte with minimal peak interference. The resulting k′ of 5.9, well above the pre-set threshold of 2, indicated a symmetric peak shape and effective retention. The Phenomenex Luna^®^ Polar Pesticides column was selected for further method development due to its enhanced analyte resolution and sensitivity.

### 3.2. Analytical Method Validation

The linearity field was first investigated with a matrix-matched calibration curve in a range from 1 µg/mL to 100 µg/mL (corresponding to 8 nmol/mL–8 µmol/mL) in PBS-BSA. The calibration settings were performed with a non-forced linear curve fit with a weighing factor of 1/x^2^. The residual accuracy of the back-calculated calibrants was ±15% of the nominal concentration and the regression coefficient R2 was >0.99. The LOQ was set at 10 µg/mL (80 nmol/mL) in whole blood and 1 µg/mL (8 nmol/mL) in plasma. In both cases the signal-to-noise ratio was over 10 and with a standard deviation lower than 15% after five analyses. The precision and the accuracy of the method were studied at four concentrations: LOQ, low (threefold LOQ concentration), medium (50% of the calibration curve) and high (75% of the calibration curve). Intra- and inter-run precision and accuracy for each level of whole blood and plasma are summarised in Table 1 and Table 2, respectively. As shown, the inter-assay accuracy was between 85.0% and 98.0% for whole blood and between 88.5% and 99.2% for plasma. The inter-assay coefficient of variation (CV) was lower than 15% in both matrices, including LOQ concentration. Matrix effect (EM) evaluations were conducted at the LOQ concentration and analysed in triplicate across three distinct analytical sessions. The data, presented in Table 1 and Table 2, indicate that for matrix effect remains within the range of 82% to 95% in whole blood and 82% to 98% in plasma. Carryover in the blank samples was lower than the 20% of the LOQ concentration after the injection of 100 µg/mL while the internal standard was below 5%.

The results of freeze-thaw stability (three cycles), short-term stability (4 h at room temperature), and long-term stability (1 week) are presented in Table 3. Taurine demonstrated satisfactory relative deviation from the nominal concentration (percentage deviation) within ±15% at three concentration levels in both plasma and whole blood.

### 3.3. Reference Interval Calculation

**Figure 2:**
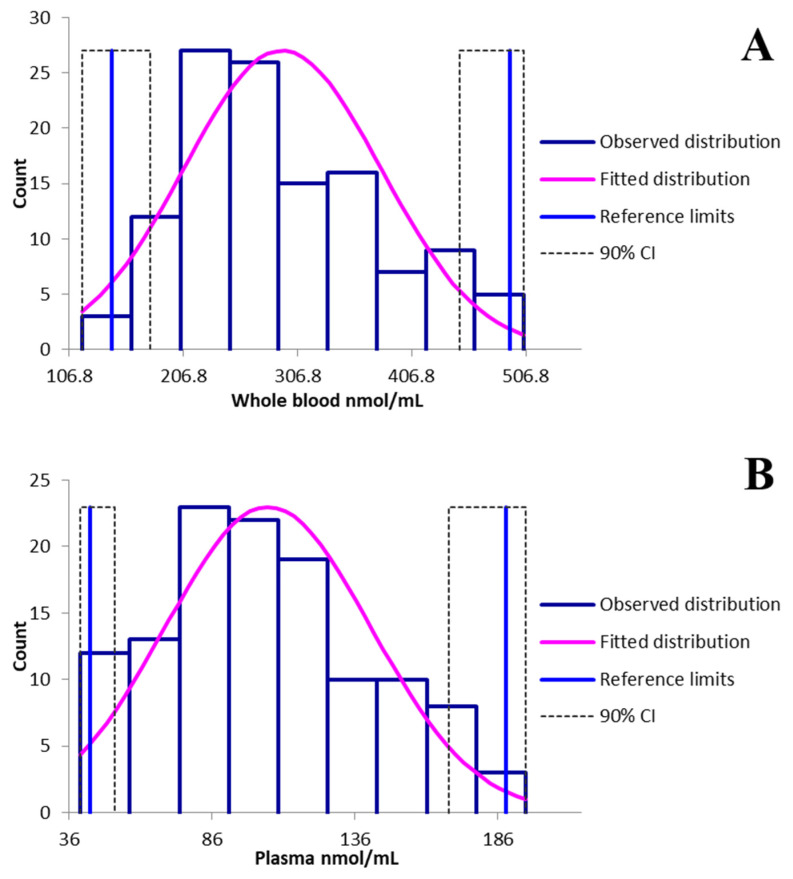
Distribution and reference intervals for taurine in whole blood (**A**) and plasma (**B**) with Reference Value Advisor (V2.1) software (Geffré et al., 2011 [30]). CI, confidence interval.

A total of 120 dogs from 41 different breeds were analysed for both whole blood and plasma matrices. This group comprised 55 males (31 intact and 24 castrated), and 65 females (15 intact and 50 spayed). The median age of the population was 8 ± 3.7 years, while the body weights ranged from 1.2 to 66 kg, with a median of 15.1 kg. Body Condition Score (BCS) using a nine-point scale (with a score of 5 as ideal) ranged from 2 to 6 (median of 4.9 ± 0.9). Causes of dog presentation included: neoplasia (13), routine check-up (15), dermatological conditions (14), oral disorders (7), pulmonary conditions (3), musculoskeletal disorders (15), neurological conditions (7), urogenital conditions (6), ophthalmological conditions (6), auditory disorders (1), foreign body ingestion (3), cardiac conditions (2), and other causes (27). To ensure our data aligned with previously published data, the reference interval values were converted to nmol/mL. The conversion was achieved by multiplying the measured concentration by 1000 and then dividing by the molecular weight of taurine (125.15 g/mol). For whole blood, both d’Agostino–Pearson and Anderson–Darling tests rejected the Gaussian distribution (*p* < 0.05 and *p* < 0.01, respectively). The mean and median corresponded to 294 and 277 nmol/mL, respectively, with a standard deviation of 86 nmol/mL. The parametric test resulted in a minimum limit of 148 nmol/mL and a maximum limit of 495 nmol/mL (Figure 2A). For plasma both d’Agostino–Pearson and Anderson–Darling tests confirmed the Gaussian distribution (*p* = 0.14 and *p* = 0.07, respectively). In this case, the mean and median corresponded to 106 nmol/mL and 104 nmol/mL, respectively, with a standard deviation of 36 nmol/mL. The parametric test yielded a minimum limit of 42 nmol/mL and a maximum limit of 183 nmol/mL (Figure 2B).

## 4. Discussion

### 4.1. Method Development and Validation

Various mobile phase compositions, including water, acetonitrile, and methanol, with or without pH modifiers (e.g., formic acid, ammonium acetate, and ammonium formate), as well as column, gradient and isocratic elution conditions, were tested to optimise chromatography. The best resolution, peak shape, and signal intensity for taurine and internal standard were achieved using a Phenomenex Luna^®^ Polar Pesticides column with water + 0.1% formic acid and methanol, at a flow rate of 0.5 mL/min. Under these conditions, analytes eluted in 2.90 min. A chromatographic run of just 10 min enabled the processing of large batches of samples in a short time. For sample preparation, several organic solvents and extraction techniques were tested, including protein precipitation with various combinations of organic solvents (methanol, acetonitrile, methanol/acetonitrile). The best recovery and background signal were obtained by applying a direct dilution with water/methanol + 0.1% formic acid. Moreover, before method validation, the recovery efficiency of the internal standard was assessed under different conditions. Results indicated that the recovery rate remained consistent when the internal standard was added directly to the first extraction solution rather than directly to the sample matrix. This finding enabled a reduction in the amount of internal standard needed in each sample, significantly lowering its concentration within the sample matrix without compromising recovery performance. As a result, the consumption of the internal standard was minimised, leading to a reduction in overall analytical costs while maintaining reliable quantification throughout the analysis. The optimised sample preparation procedure allowed us to avoid more complex and expensive extractions, such as solid-phase extraction and enabled the preparation of 20 samples in just 1 h. Due to the sensitivity of the proposed method, derivatisation agents, which are typically costly and complicate sample preparation, were not required.

Previous studies validated taurine quantification via LC-MS/MS, yet these methods commonly employed reverse-phase columns, which often resulted in limited retention times for the analyte. Additionally, the absence of a homologous internal standard in these approaches hindered accuracy, quantitation precision, and the robustness of the bioanalytical method [25,26]. A viable alternative involves the use of specialised columns designed to enhance the retention of polar compounds, achieved through hydrophilic interaction liquid chromatography (HILIC) coupled with mass spectrometry [27]. Both [25,26], used a conventional Agilent Zorbax SB-Aq reverse-phase column (100 × 2.1 mm, 3.5 µm) with a flow rate of 0.3 mL/min, achieving a retention time of approximately 1 min for the analyte and a k′1 of about 1.4, considerably lower than ours. Furthermore, these studies focused exclusively on taurine analysis in plasma, substituting the deuterated internal standard with sulfanilic acid, an analog that may introduce some degree of data uncertainty. Their limit of quantification (LOQ) was reported at 2 µg/mL, double the sensitivity we achieved. Alternatively, Awwad et al. [27] used an Acquity BEH HILIC column (100 × 2.1 mm, 1.7 µm) and employed deuterated taurine as an internal standard for analysing taurine in both urine and plasma. While their chromatographic runtime was shorter than that reported in our study and had a similar retention time, a notable limitation was their use of a solvent-based calibration curve instead of the matrix-based curve we recommend. Additionally, their extraction process was minimal, using a 1:3 dilution with an acetonitrile/methanol mixture for plasma and 1:15 for urine. Such limited dilution may not sufficiently clean the sample before injection, potentially impacting the longevity of the chromatographic column and overall analytical quality. With our greater instrumental sensitivity, we achieve higher sample dilution, enhancing the preservation of both instrumentation and data integrity.

### 4.2. Dog Whole Blood and Plasma Analysis

Various groups have undertaken the task of establishing taurine ranges in dogs; however, these efforts often focus on a single breed, a specific pathological context, or a specific diet [20,22,25,32,33,34]. A reference interval typically represents the range of values that include the central 95% of measurements from a healthy population for a particular parameter. These intervals are calculated using statistical methods and serve as essential guidelines for interpreting test results. When a test result falls within the reference interval, it is generally considered normal. However, results outside this range might indicate potential physiological or pathological changes, prompting further evaluation to determine their significance. One of the distinctive elements of the article we propose is that we have considered an extensive population of samples to ensure robust statistics applied to an effective analysis method. Overall, the intervals we propose tend to align with those previously defined in the literature [14]. Specifically, for both whole blood and plasma, the average values are similar to those described. Notably, the main differences are observed in whole blood, where the upper and lower limits tend to be more extensive. This could be attributed to the greater variability of the data, stemming from the larger number of subjects analysed and the inclusion of a wider variety of breeds. Furthermore, our analytical method was previously validated using dog serum and has since been applied by other research groups to investigate the role of this analyte in the diet of Golden Retrievers and their predisposition to dilated cardiomyopathy [35,36]. We subsequently opted to replace serum with plasma and validated this adjustment, as it is well-documented that a minor release of taurine from platelets during the blood coagulation process in serum formation may lead to overestimated values [37]. The newly established reference intervals (RIs) for taurine, determined through LC-MS/MS analysis, have implications for clinical diagnosis, nutritional guidance, and treatment strategies in dogs. By encompassing a broader and more diverse population, these RIs provide a representative benchmark for the general canine population, surpassing the limitations of breed-specific RIs deriving from experimental studies using a small sample size [20,25,32,33,34]. For clinicians, these updated RIs serve as a more reliable tool for identifying taurine deficiencies or abnormalities, which are particularly critical for conditions such as dilated cardiomyopathy. The advanced precision of the LC-MS/MS methodology ensures accurate detection of even subtle deviations in taurine concentrations, facilitating earlier diagnosis and timely intervention. From a nutritional perspective, the detection of dogs with depleted taurine concentrations enables more precise dietary recommendations for commercial, home-prepared diets, and taurine supplements. With more accurate RIs, veterinarians and nutritionists can therefore design tailored supplementation strategies to optimise taurine levels, avoiding the risks of both deficiency and unnecessary over-supplementation.

Our research does not aim to replace existing reference intervals but rather to complement them, enriching the research with data obtained through a particularly reliable and validated methodology such as LC-MS/MS. The novelty of this work lies in the method’s robustness, which contributed to more accurate and precise taurine quantification in dog whole blood and plasma, and the determination of RIs within a substantial population of dogs. This advancement in methodology enhances the overall understanding of taurine dynamics and facilitates more accurate assessments in the context of canine health.

## 5. Conclusions

The newly developed method offers a practical and efficient solution, providing a low-cost and time-saving alternative to current approaches. Following successful validation, it was first applied to 120 whole blood and plasma samples from healthy dogs, or to dogs for which medical attention had been sought for pathologies unrelated to the gastrointestinal tract, enabling the determination of RIs in this population. This easy-to-implement technique holds promise for future research into the link between taurine concentrations and various diseases, including chronic conditions like dilated cardiomyopathy and retinal degeneration. Moreover, it could be used to monitor taurine concentration in dogs requiring dietary supplementation.

## Figures and Tables

**Table 1:** Accuracy of the determination of taurine in whole blood, in terms of mean detected concentration (µg/mL). CV acceptance criteria for LOQ: ≤20%; CV acceptance for low, medium, high levels ≤ 15%; EM% acceptance criteria: ≤20%.

Spike Level		Intra-Assay-1	Intra-Assay-2	Intra-Assay-3	Inter-Assay
LOQ	Mean	10.9	9.14	9.36	9.25
(10 µg/mL)	CV	14.1	13.0	10.6	13.4
	Accuracy	108.9	91.4	93.6	98.0
Low	Mean	25.6	27.5	25.6	26.2
(30 µg/mL)	CV	3.40	9.36	8.06	6.99
	Accuracy	85.4	91.6	85.2	87.4
Medium	Mean	43.0	43.9	43.4	43.4
(50 µg/mL)	CV	0.97	6.12	0.77	2.63
	Accuracy	86.0	87.7	86.8	86.8
High	Mean	66.2	64.1	64.3	63.8
(75 µg/mL)	CV	5.07	3.50	1.10	2.63
	Accuracy	88.3	85.5	81.4	85.0
EM %	Mean	95.0	82.7	91.7	
	CV	6.99	1.90	4.14	

Accuracy (%) = expressed as [(mean observed concentrations − nominal concentration)/(nominal concentration)] × 100); CV (%) = coefficient of variation: (standard deviation/mean) × 100. LOQ, limit of quantification; EM, Matrix Effect.

**Table 2:** Accuracy of the determination of taurine in plasma, in terms of mean detected concentration (µg/mL). CV acceptance criteria for LOQ: ≤20%; CV acceptance for low, medium, high levels ≤ 15%; EM% acceptance criteria: ≤20%.

Spike Level		Intra-Assay-1	Intra-Assay-2	Intra-Assay-3	Inter-Assay
LOQ	Mean	0.90	1.08	0.97	0.98
(1 µg/mL)	CV	13.1	8.87	5.12	8.94
	Accuracy	90.5	107.6	96.8	98.3
Low	Mean	2.58	3.24	3.10	2.98
(3 µg/mL)	CV	4.93	6.38	9.88	7.18
	Accuracy	86.1	108.1	103.4	99.2
Medium	Mean	42.7	43.7	46.7	44.3
(50 µg/mL)	CV	3.11	1.69	1.62	2.12
	Accuracy	85.4	86.7	93.5	88.5
High	Mean	69.7	64.9	69.6	67.7
(75 µg/mL)	CV	5.26	3.23	2.19	3.64
	Accuracy	92.9	86.5	92.8	90.7
EM %	Mean	98.8	82.5	83.0	
	CV	0.35	0.56	3.98	

Accuracy (%) = expressed as [(mean observed concentrations − nominal concentration)/(nominal concentration)] × 100); CV (%) = coefficient of variation: (standard deviation/mean) × 100. LOQ, limit of quantification; EM, Matrix Effect.

**Table 3:** The results of freeze-thaw stability (three cycles), short-term stability (4 h at room temperature), and long-term stability (1 week).

Sample Type	Stability Type	Nominal Concentration, µg/mL	Mean, µg/mL	Standard Deviation, µg/mL	Percentage Deviation
Plasma	Freeze-thaw	3	2.91	0.08	−3.11
		30	30.5	0.45	1.57
		50	50.6	1.67	1.23
	Short term	3	2.58	0.08	−14.0
		30	26.2	0.10	−12.6
		50	43.1	0.14	−13.8
	Long term	3	2.90	0.11	3.65
		30	29.3	0.27	0.92
		50	48.2	1.03	2.13
Whole blood	Freeze-thaw	30	28.4	0.53	−5.28
		50	49.4	0.52	−1.22
		75	76.8	6.23	2.40
	Short term	30	28.2	1.36	−5.88
		50	47.2	0.48	−5.52
		75	70.3	0.23	−6.30
	Long term	30	30.2	0.81	2.67
		50	50.1	0.79	1.57
		75	74.5	0.26	0.35

## Data Availability

Dataset available on request from the authors.
